# Prognostic values of right ventricular echocardiography functional parameters for mortality prediction in precapillary pulmonary hypertension: a systematic review and meta-analysis

**DOI:** 10.1186/s43044-024-00539-6

**Published:** 2024-08-14

**Authors:** Bryan Gervais de Liyis, Luh Oliva Saraswati Suastika, Jane Carissa Sutedja, Gusti Ngurah Prana Jagannatha, Anastasya Maria Kosasih, Alif Hakim Alamsyah

**Affiliations:** 1 Faculty of Medicine, Universitas Udayana, Prof. I.G.N.G Ngoerah General Hospital, Denpasar, Bali Indonesia; 2 Department of Cardiology and Vascular Medicine, Faculty of Medicine, Universitas Udayana, Prof. I.G.N.G Ngoerah General Hospital, Diponegoro Street, Dauh Puri Klod, Denpasar, Bali Indonesia

**Keywords:** Echocardiography, Mortality, Precapillary pulmonary hypertension, Prognosis, Right ventricle

## Abstract

**Background:**

Echocardiographic prognostic indicators of precapillary pulmonary hypertension (PH) mortality has been inconclusive. This study aims to examine the prognostic values of right ventricular echocardiographic functional parameters in predicting precapillary PH mortality.

**Methods:**

Systematic searches were conducted in the ScienceDirect, Medline, and Cochrane databases for longitudinal studies. Assessments included means and hazard ratios (HRs) for Tricuspid Annular Plane Systolic Excursion (TAPSE), Right Ventricular Systolic Pressure (RVSP), Right Ventricular Longitudinal Strain (RVLS), Right Ventricular Fractional Area Change (RVFAC), Right Ventricular Ejection Fraction (RVEF), and Right Ventricular Index of Myocardial Performance (RIMP).

**Results:**

The meta-analysis included 24 cohort studies comprising 2171 participants. Mean values were as follows: TAPSE 17.62 mm, RVSP 77.50 mmHg, RVLS − 16.78%, RVFAC 29.81%, RVEF 37.56%, and RIMP 0.52. TAPSE (HR: 1.28; 95% CI 1.17–1.40; *p* < 0.001), RVLS (HR: 1.74; 95% CI 1.34–2.26; *p* < 0.001), RVFAC (HR: 1.40; 95% CI 1.13–1.75; *p* < 0.001), RVEF (HR: 1.08; 95% CI 1.02–1.15; *p* = 0.01), and RIMP (HR: 1.51; 95% CI 1.23–1.86; *p* < 0.001) emerged as significant prognosticators of precapillary PH mortality, with the exception of RVSP (HR: 1.04; 95% CI 0.99–1.09; *p* = 0.14). TAPSE summary receiver operating characteristics (sROC) analysis yielded an area under the curve (AUC) of 0.85 [95% CI 0.81–0.88] with a sensitivity of 0.81 [95% CI 0.63–0.91] and a specificity of 0.74 [95% CI 0.54–0.87]. RVLS sROC resulted in an AUC of 0.74 [95% CI 0.70–0.78] with a sensitivity of 0.74 [95% CI 0.57–0.86] and a specificity of 0.69 [95% CI 0.64–0.75].

**Conclusions:**

TAPSE, RVLS, RVFAC, RVEF, and RIMP demonstrated promise as valuable prognostic indicators for precapillary PH mortality.

**Supplementary Information:**

The online version contains supplementary material available at 10.1186/s43044-024-00539-6.

## Background

According to the 2022 guidelines of the European Society of Cardiology (ESC) and the European Respiratory Society (ERS) for the diagnosis and treatment of pulmonary hypertension (PH), precapillary PH is defined by a mean pulmonary artery pressure (mPAP) exceeding 20 mmHg, a pulmonary artery wedge pressure (PAWP) of 15 mmHg or less, and a pulmonary vascular resistance (PVR) greater than 2 Wood units (WU) [1]. Precapillary PH encompasses the following clinical groups: Group 1 (comprising pulmonary arterial hypertension (PAH) associated with idiopathic, heritable, drug-induced, connective tissue diseases, and congenital heart diseases), Group 3 (comprising PH associated with lung diseases and/or hypoxia), Group 4 (pulmonary hypertension associated with chronic pulmonary artery obstruction), and Group 5 (comprising those with unclear and multifactorial mechanisms). Postcapillary PH, on the other hand, falls under clinical Group 2, signifying PH stemming from left heart diseases. The incidence of precapillary PH is approximately 6 cases per million adults, with a prevalence ranging from about 49 to 55 cases per million adults [2]. The mean age across studies varied between 43 and 67 years, with a predominant female gender representation ranging from 55 to 81% [3]. In recent times, there is mounting evidence, indicating that PH frequently occurs as a complication of several prevalent diseases. Importantly, the development of pulmonary hypertension in these contexts is consistently linked to the worsening of clinical symptoms and heightened mortality rate. A study demonstrated that the 15-year survival rates were 59.1% for precapillary pulmonary hypertension, 68.5% for PAH, and 44.3% for chronic thromboembolic pulmonary hypertension (CTEPH) [4].

Numerous echocardiographic indices have been suggested as potential prognostic markers in individuals with PH. Nevertheless, the majority of these indices have been derived from various heart chambers, encompassing heterogeneous groups of patients with distinct PH etiologies, insufficient assessment periods, and limited sample sizes [5]. The right ventricle (RV), the cardiac chamber directly impacted by PH, is predominantly absent from most severity classification schemes. This phenomenon may be attributed to the inherent complexity in echocardiographic assessment of the right heart, primarily owing to the intricate morphology of the RV and its physiology, which is influenced by load-dependent factors [6]. There presently exists no universally recognized standard for assessing the RV functional parameters within the realm of prognostic echocardiographic examinations [7]. In the context of PH, the echocardiographic characteristics and behavior of the RV are frequently overlooked by attending physicians, resulting in the omission of valuable and consistent data.

While awareness, diagnosis, and clinical management of precapillary PH have greatly improved over the past few decades, there is still a lack of consensus in the scientific community on which estimates could reflect the prognosis. In essence, this meta-analysis addresses the absence of mortality prediction tools in precapillary PH, offering valuable RV echocardiographic insights that can significantly enhance patient care and outcomes in this clinical context.

## Methods

### Study design and inclusion criteria

This meta-analysis strictly adhered to the PRISMA guidelines, ensuring a methodical and comprehensive approach. The study protocol was registered and approved in advance in the PROSPERO database (ID: CRD42023461510) before initiating the systematic search. The inclusion criteria were meticulously defined, focusing on both retrospective and prospective cohort studies that specifically examined the prognostic value of RV functional parameters in patients with precapillary PH. The entire process, from conducting the literature search to extracting data and assessing potential biases, was carried out by two authors. Any discrepancies or uncertainties regarding the eligibility of studies were thoughtfully resolved through consensus, involving an additional author in the decision-making process.

The inclusion criteria comprised several key aspects: The studies had to evaluate at least one prognostic parameter, involve patients with mean age at least 40 years, follow up with patients for no less than 6 months, and perform echocardiographic assessments using three-dimensional speckle tracking echocardiography within 1 week of diagnosing precapillary PH. PH participants incorporated into the research were exclusively categorized within groups 1, 3, and 4. Group 5 PH was omitted due to its inclusive nature, encompassing both precapillary and postcapillary forms, typically characterized by multifactorial etiologies. Studies with less than a 6-month follow-up duration, those involving pediatric populations, participants who had undergone chemotherapy, individuals with autoimmune or psychiatric conditions, and studies lacking data on hazard ratios (HRs) were excluded from the analysis. Moreover, studies focusing on patients with group 2 PH were excluded due to its postcapillary nature, ensuring a more homogeneous study population. Furthermore, ethical approval for patient enrollment at all participating sites was obtained from the relevant institutional review boards.

### Literature search and selection

**Fig. 1:**
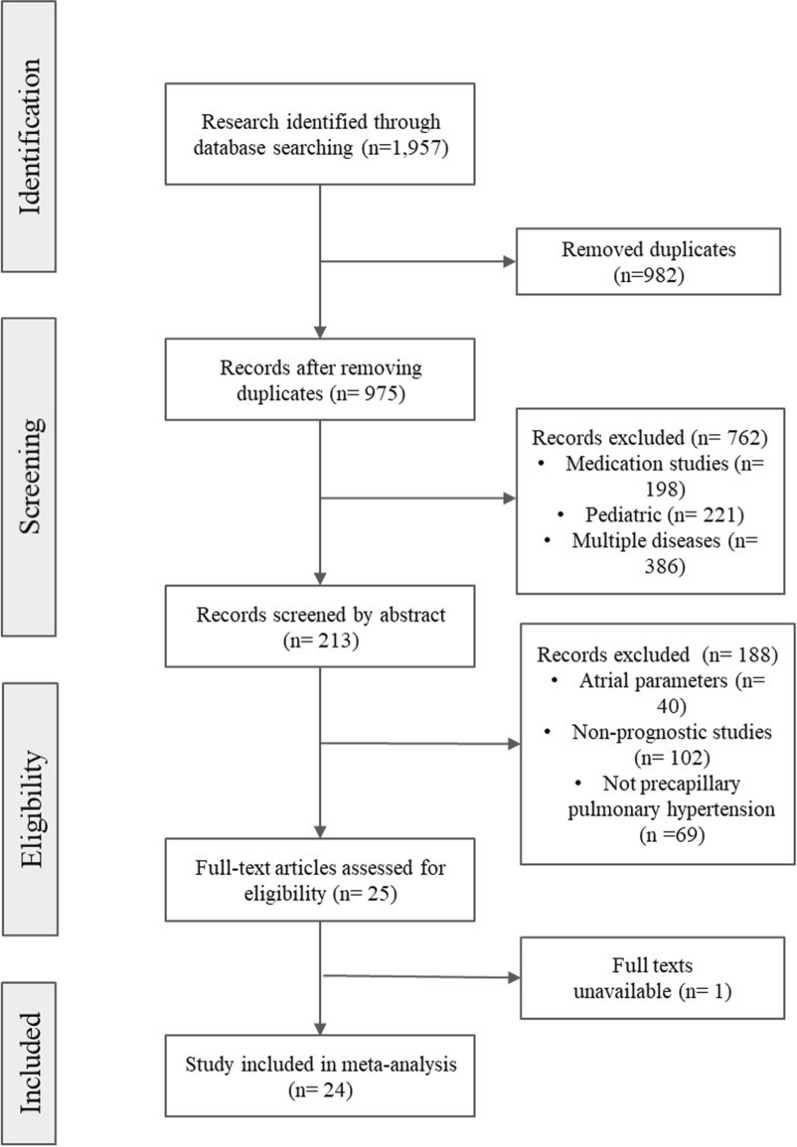
PRISMA flowchart

A comprehensive and systematic literature search was carried out, employing the ScienceDirect, Medline, and Cochrane databases, spanning from January 2002 to December 2023. Language restrictions were intentionally omitted during the search process. The search strategy encompassed both Medical Subject Headings (MeSH) terms and pertinent free-text keywords germane to the subject of inquiry. From the initial pool of 1,957 retrieved manuscripts, a total of 213 conformed to the pre-established inclusion criteria, as delineated in the PRISMA flowchart (Fig. 1). Additionally, a meticulous manual examination of the references within the identified studies was conducted to ensure the comprehensiveness of the search and to identify any ancillary literature germane to the subject matter. Ultimately, 24 studies were deemed suitable for incorporation into the quantitative analysis [1–24].

### Quality assessment of included studies

A thorough evaluation of potential bias was systematically conducted employing the cohort subscale of the Newcastle–Ottawa Scale (NOS) (Supplementary File 1), encompassing nine critical components. This comprehensive tool evaluates various facets of each study, comprising but not limited to selection bias, comparability bias, and outcome bias. This multifaceted approach took into account pivotal considerations, including the study design, limitations, consistency, directness, and precision of the studies, as well as the potential risk of publication bias.

### Data extraction

A thorough and systematic data extraction process was meticulously executed to compile comprehensive demographic, baseline characteristics, and outcome-related data from the included studies. This process entailed a detailed assessment of key parameters, encompassing the country of origin for each study, the total study population, mean age, percentage of female participants, New York Heart Association (NYHA) functional class (FC), World Health Organization (WHO) FC, 6-min walking distance (6MWD), mean pulmonary arterial pressure (mPAP), and the duration of follow-up periods. The research outcomes were predicated on pivotal indicators, specifically, the means and hazard ratios (HRs) of various RV functional parameters, including Tricuspid Annular Plane Systolic Excursion (TAPSE), Right Ventricular Systolic Pressure (RVSP), Right Ventricular Longitudinal Strain (RVLS), Right Ventricular Fractional Area Change (RVFAC), Right Ventricular Ejection Fraction (RVEF), as well as the Right Ventricular Index of Myocardial Performance (RIMP).

A pooled analysis of summary receiver operating characteristics (sROC) curves was conducted, yielding the pooled area under the curve (AUC), sensitivity (%), and specificity (%). Additionally, Kaplan–Meier survival analysis was performed to assess mortality trends over time. Hazard ratio subgroup analyses were carried out based on specific criteria, including NYHA FC (III/IV) ≥ 50%, NYHA FC (III/IV) < 50%, WHO FC (III/IV) ≥ 50%, WHO FC (III/IV) < 50%, Group 1 PH (PH) exclusively, Group 3 and 4 PH, mPAP ≥ 50 mmHg, and mPAP < 50 mmHg subgroups for all six assessed RV functional parameters.

### Data synthesis and analysis

A rigorous statistical approach ensured precise analysis. Continuous data were expressed as mean ± SD, categorical variables as absolute numbers or percentages. Data presentation, including 95% CIs, facilitated inter-study comparisons. The REML model amalgamated effect estimates, considering treatment effect variations. The Mantel–Haenszel method assessed heterogeneity, guiding model selection. Visual representation used forest, funnel, and Galbraith plots (Supplementary File 1). Stata software version 18 (StataCorp, College Station, TX, USA) conducted analyses with *p* < 0.05 significance. Rigorous statistics provided a nuanced exploration, contributing to evidence-based synthesis. Cumulative incidence curves depicted significant echocardiographic measures. Significant measures underwent sROC curve analysis for AUCs, sensitivity, and specificity.

## Results

### Study selection

The search yielded 1,957 articles, with 982 duplicates. After title and abstract screening, exclusions included medication studies (*n* = 982), pediatric-focused (*n* = 221), and those addressing multiple diseases (*n* = 386). Among the 213 remaining studies, 40 discussed atrial echocardiographic parameters, 102 lacked prognostic assessment, and 69 were irrelevant to precapillary PH. Full-text screening of the remaining 25 studies resulted in the inclusion of 24 studies in the meta-analysis (Fig. 1).

### Characteristics and quality assessment of included studies

The twenty-four cohort studies encompassed in this meta-analysis span the period between 2002 and 2023. These studies were conducted across a diverse array of countries, including Australia, Belgium, Brazil, China, France, Germany, Greece, Italy, Japan, the Netherlands, Romania, and the USA. Detailed patient characteristics for each of the cohort studies are presented in Table 1. The cumulative study population consisted of 2,171 patients, exhibiting an average age of 51.18 ± 14.16 years. Among these patients, women constituted 70.64%. The mean 6MWD and mPAP across the various studies were 366.07 ± 119.98 m and 51.90 ± 14.09 mmHg, respectively. Although the duration of follow-up exhibited variation among the studies, ranging from 6 to 50 months, the mean follow-up period approximated 30.42 ± 12.83 months.

**Table 1:** Demographic and clinical characteristics of the study populations

Study, year	Country	Total population (*n*)	Mean age (years)	Female (%)	NYHA FC I (%)	NYHA FC II (%)	NYHA FC III (%)	NYHA FC IV (%)	WHO FC I (%)	WHO FC II (%)	WHO FC III (%)	WHO FC IV (%)	6MWD (m)	mPAP (mmHg)	Follow-up (months)
Badagliacca et al., 2016 [8]	Italy	102	52 ± 14	61					2.7 ± 0.4				430 ± 6	48 ± 15	17.36 ± 9.99
Butcher et al. 2022 [9]	Greece	51	58 ± 13	69										41 ± 3	35
Chen et al., 2017 [10]	China	40	43 ± 14	75					5	10	62.5	22.5	347 ± 121	60 ± 9	25.08 ± 8.35
Ciarka et al., 2010 [11]	Belgium	32	53 ± 16	65.62	3	6	50	41					375 ± 118	55 ± 13	20.6
da Costa et al., 2017 [12]	Brazil	66	45 ± 14	80	67		33						431 ± 92	59 ± 14	46.8
Dandel et al., 2014 [13]	Germany	79	45.67 ± 9.46	48.08					0	2.53	97.47	0	302.67 ± 50		92
Fine et al., 2013 [14]	China	406	59.4 ± 16.0	65.02					20	34	38	8	363.5 ± 246.92	32.7 ± 25.29	16.4
Forfia et al., 2006 [15]	USA	63	55 ± 15	82.54					30		70			45 ± 12	19.3
Ghio et al., 2010 [16]	Italy	59	46.4 ± 16.1	62.71						33.90	59.32	6.78		54.5 ± 14.7	52
Giusca et al., 2013 [17]	Romania	32	39 ± 15	68.75	0	40.6	56.2	3.2					331.8 ± 148	58 ± 19.5	14
Greiner et al., 2018 [18]	Germany	65	63.5 ± 14.8	66					5.00	32.00	49.00	14.00			27.74
Haddad et al., 2015 [19]	USA, the Netherlands	95	43 ± 11	79	14.74		85.26						432 ± 117	54 ± 14	50.4 ± 38.4
Haeck et al., 2012 [20]	The Netherlands	142	59.15 ± 22.28	62.68	2.5 ± 1										31.2
Hulshof et al., 2021 [21]	The Netherlands	143	61 ± 16	70					81.82	0	0	18.18			60
Ishii et al., 2023 [22]	Japan	34	29.0 ± 10.3	71					0	0	94.12	5.88	406.75 ± 195.77	49.69 ± 6.63	30.72
Li et al., 2021 [23]	China	203	49.2 ± 14.5	71.92					9.85	64.04	26.11		355.54 ± 100.55	47.9 ± 13.5	20.9
Mahapatra et al., 2006 [24]	USA	54	52 ± 11	75.93					0	24	52	24	307 ± 87	52 ± 14	49.25 ± 3.55
Mathai et al., 2011 [25]	USA	50	61 ± 11	98	4	26	60	10					325 ± 88	42 ± 10	15.7
Moceri et al., 2017 [26]	France	104	65.57 ± 1.46	55.77					0	38	46	20	359.5 ± 106	43.3 ± 12.6	6.7
Murata et al., 2016 [27]	Japan	86	50 ± 17	73.26					18.60	47.67	33.72	0	378 ± 102	34 ± 12	13.91
Park et al., 2015 [28]	USA	51	48 ± 14	78.43	4	61				61	35		411 ± 101	53.5 ± 18.7	45 ± 15
Raymond et al., 2002 [29]	USA	81	40 ± 15	73			74	26					294.44 ± 125.88	60 ± 13	6
Sachdev et al., 2011 [30]	USA	80	56 ± 14	76					28		63	9	332 ± 129	50 ± 10	27.9 ± 1.7
van Kessel et al., 2016 [31]	Australia	53	54.48 ± 25	66.04					41.51	41.51	9.43	7.55	407.1 ± 225.64	98.5 ± 32.0	6

NHYA, New York Heart Association; FC, functional class; WHO, World Health Organization

Among the selected studies, four of them incorporated patients presenting with NYHA FC III/IV > 50% [11, 17, 19, 29]. In contrast, eight studies encompassed patients with WHO FC III/IV > 50% [10, 13, 16, 18, 22, 24, 26, 30]. Regarding the classification of precapillary hypertension, ten studies exclusively included patients diagnosed with Group I PH [8, 10–13, 16, 19, 26, 28, 30]. This category encompassed etiologies related to inheritable causes, medication usage, and connective tissue diseases. Furthermore, ten studies featured patients with mPAP ≥ 50 mmHg [10–12, 16, 17, 19, 24, 28–30] in their cohorts. In Supplementary File 1, we provide a summary of our quality assessment. The individual NOS scores within this cohort ranged from 7 to 8.

### Mean and hazard ratios of right ventricular functional parameters

**Fig. 2:**
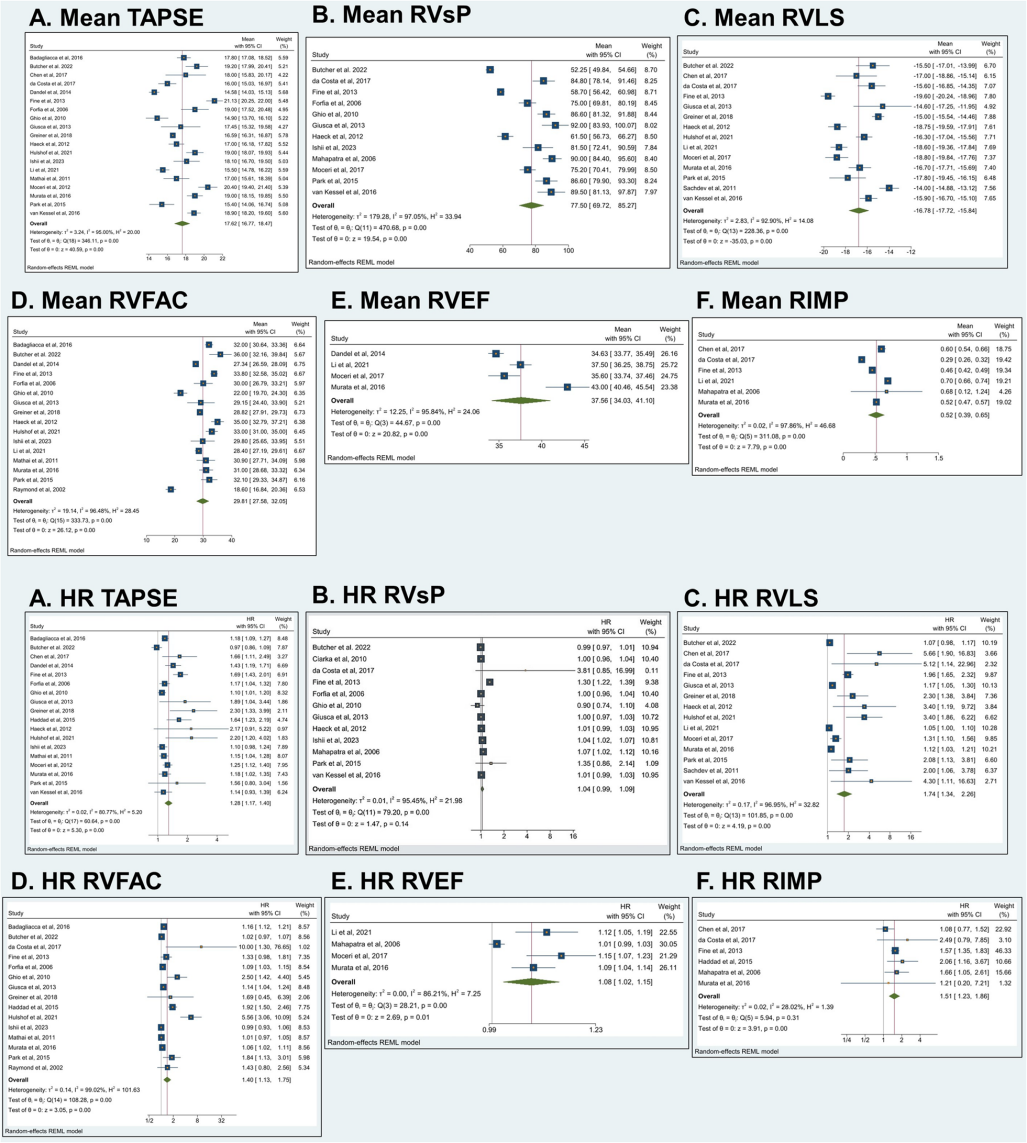
Mean values and hazard ratios of right ventricular functional parameters. **A** TAPSE, **B** RVSP, **C** RVLS, **D** RVFAC, **E** RVEF, **F** RIMP. Abbreviations: 95% CI, 95% confidence interval; HR, hazard ratio; RIMP, Right Ventricular Index of Myocardial Performance; RVFAC, Right Ventricular Fractional Area Change; RVEF, Right Ventricular Ejection Fraction; RVLS, Right Ventricular Longitudinal Strain; RVSP, Right Ventricular Systolic Pressure; TAPSE, Tricuspid Annular Plane Systolic Excursion

Table 2 displays the mean values and HRs for RV functional parameters concerning mortality in precapillary PH. The pooled mean values were as follows: TAPSE at 17.62 [16.77–18.47] mm, RVSP at 77.50 [69.72–85.27] mmHg, RVLS at − 16.78 [− 17.72 to − 15.84]%, RVFAC at 29.81 [27.58–32.05]%, RVEF at 37.56 [34.03–41.10]%, and RIMP at 0.52 [0.39–0.65] (Fig. 2). Despite the presence of high heterogeneity, all these values were determined to be statistically significant.

**Table 2:** Mean values of right ventricular functional parameter analysis in precapillary pulmonary hypertension

RV functional parameter	No. of studies (mean)	Mean [95% CI]	*p* value	*I*^2^%	No. of studies (HR)	HR [95% CI]	*p* value	*I*^2^%
TAPSE (mm)	19	17.62 [16.77–18.47]	< 0.001*	95.00	18	1.28 [1.17–1.40]	< 0.001*	80.77
RVSP (mmHg)	12	77.50 [69.72–85.27]	< 0.001*	97.05	11	1.04 [0.99–1.09]	0.14	95.45
RVLS (%)	14	− 16.78 [− 17.72 to − 15.84]	< 0.001*	92.90	14	1.74 [1.34–2.26]	< 0.001*	96.95
RVFAC (%)	16	29.81 [27.58–32.05]	< 0.001*	96.48	15	1.40 [1.13–1.75]	< 0.001*	99.02
RVEF (%)	4	37.56 [34.03–41.10]	< 0.001*	95.84	4	1.08 [1.02–1.15]	0.01*	86.21
RIMP	6	0.52 [0.39–0.65]	< 0.001*	97.86	6	1.51 [1.23–1.86]	< 0.001*	28.02

TAPSE, Tricuspid Annular Plane Systolic Excursion; RVSP, Right Ventricular Systolic Pressure; RVLS, Right Ventricular Longitudinal Strain; RVFAC, Right Ventricular Fractional Area Change; RVEF, Right Ventricular Ejection Fraction; RIMP, Right Ventricular Index of Myocardial Performance

*Shows significance of < 0.05

Concerning prognostic significance, the following parameters were identified as significant prognostic markers for mortality in precapillary PH: TAPSE (HR: 1.28; 95% CI 1.17–1.40; *p* < 0.001), RVLS (HR: 1.74; 95% CI 1.34–2.26; *p* < 0.001), RVFAC (HR: 1.40; 95% CI 1.13–1.75; *p* < 0.001), RVEF (HR: 1.08; 95% CI 1.02–1.15; *p* = 0.01), and RIMP (HR: 1.51; 95% CI: 1.23–1.86; *p* < 0.001). In contrast, RVSP (HR: 1.04; 95% CI 0.99–1.09; *p* = 0.14) was not identified as a prognostic marker of mortality (Fig. 2). Additionally, the HRs for TAPSE, RVFAC, and RVEF were inadvertently inverted during the data analysis process. Consequently, lower values of these parameters are unequivocally linked to a higher mortality risk within the context of precapillary PH. This rectification shows the inverse relationship that exists, providing a clearer understanding of the prognostic implications associated with these critical RV functional parameters.

Subgroup analyses, delineated in accordance with the aforementioned criteria, have unveiled significant insights (Table 3). While the overall prognostic potential of RVSP for mortality remains inconclusive, an intriguing exception has come to light among patients primarily classified under WHO FC III/IV (HR: 1.05; 95% CI 1.03–1.07; *p* < 0.001), where RVSP emerged as a noteworthy prognostic factor for mortality. Furthermore, RVFAC has exhibited augmented prognostic significance within subgroups characterized by WHO FC III/IV < 50% (HR: 1.40; 95% CI 1.10–1.78; *p* < 0.01), Group 1 PH (HR: 1.78; 95% CI 1.25–2.55; *p* < 0.001), and mPAP ≥ 50 mmHg (HR: 1.70; 95% CI 1.25–2.30; *p* < 0.001). Similarly, both RVEF and RIMP have demonstrated heightened prognostic capacity within the subgroup characterized by WHO FC III/IV < 50% (HR: 1.10; 95% CI 1.06–1.14; *p* < 0.001 and HR: 1.58; 95% CI 1.36–1.84; *p* < 0.001, respectively). RIMP exhibited superior predictive capabilities in the context of mortality within Group 3 and 4 PH (HR: 1.58; 95% CI 1.36–1.82; *p* < 0.001). Notably, the predictive value of RVEF for mortality in groups stratified by etiology and mPAP remains inconclusive due to limited data availability, with only one study analyzed for each group.

**Table 3:** Prognostic values of right ventricular functional parameter analysis in precapillary pulmonary hypertension

RV functional parameter	No. of studies	HR [95% CI]	*p* value	*I*^2^%
**TAPSE (mm)**	**18**	**1.28 [1.17**–**1.40]**	< 0.001*	**80.77**
NYHA FC III/IV ≥ 50%	2	1.68 [1.30–2.18]	< 0.001*	0
NYHA FC III/IV < 50%	16	1.25 [1.14–1.36]	< 0.001*	79.34
WHO FC III/IV ≥ 50%	6	1.25 [1.11–1.47]	< 0.001*	78.50
WHO FC III/IV < 50%	12	1.29 [1.14–1.46]	< 0.001*	82.60
Group 1 PH only	7	1.29 [1.15–1.44]	< 0.001*	72.26
Group 3 and 4 PH	11	1.29 [1.12–1.49]	< 0.001*	85.07
mPAP ≥ 50 mmHg	13	1.25 [1.13–1.37]	< 0.001*	80.68
mPAP < 50 mmHg	5	1.44 [1.13–1.85]	< 0.001*	63.04
**RVSP (mmHg)**	**11**	**1.04 [0.99**–**1.09]**	**0.14**	**95.45**
NYHA FC III/IV ≥ 50%	2	1.00 [0.98–1.02]	1	0.13
NYHA FC III/IV < 50%	9	1.05 [0.98–1.12]	0.14	96.48
WHO FC III/IV ≥ 50%	3	1.05 [1.03–1.07]	< 0.001*	0.01
WHO FC III/IV < 50%	8	1.04 [0.98–1.12]	0.21	96.98
Group 1 PH only	4	1.00 [0.96–1.04]	0.96	0
Group 3 and 4 PH	7	1.05 [0.99–1.11]	0.13	97.17
mPAP ≥ 50 mmHg	6	1.02 [0.98–1.06]	0.38	50.79
mPAP < 50 mmHg	5	1.05 [0.97–1.14]	0.22	98.21
**RVLS (%)**	**14**	**1.74 [1.34**–**2.26]**	< 0.001*	**96.95**
NYHA FC III/IV ≥ 50%	1	1.17 [1.05–1.30]	< 0.001*	–
NYHA FC III/IV < 50%	13	1.84 [1.39–2.45]	< 0.001*	96.90
WHO FC III/IV ≥ 50%	4	2.04 [1.25–3.35]	< 0.001*	72.52
WHO FC III/IV < 50%	10	1.65 [1.21–2.26]	< 0.001*	97.83
Group 1 PH only	6	1.84 [1.21–2.81]	< 0.001*	88.63
Group 3 and 4 PH	8	1.72 [1.20–2.46]	< 0.001*	98.14
mPAP ≥ 50 mmHg	5	2.17 [1.24–3.79]	0.01*	75.41
mPAP < 50 mmHg	9	1.62 [1.20–2.20]	< 0.001*	97.58
**RVFAC (%)**	**15**	**1.40 [1.13**–**1.75]**	< 0.001*	**99.02**
NYHA FC III/IV ≥ 50%	3	1.44 [1.01–2.07]	0.05*	83.68
NYHA FC III/IV < 50%	12	1.43 [1.07–1.90]	0.01*	99.40
WHO FC III/IV ≥ 50%	3	1.51 [0.77–2.95]	0.23	77.45
WHO FC III/IV < 50%	12	1.40 [1.10–1.78]	0.01*	99.14
Group 1 PH only	5	1.78 [1.25–2.55]	< 0.001*	82.35
Group 3 and 4 PH	10	1.26 [0.98–1.62]	0.07	99.11
mPAP ≥ 50 mmHg	6	1.70 [1.25–2.30]	< 0.001*	75.37
mPAP < 50 mmHg	9	1.26 [0.95–1.67]	0.11	99.46
**RVEF (%)**	**4**	**1.08 [1.02**–**1.15]**	0.01*	**86.21**
NYHA FC III/IV ≥ 50%	–	–	–	–
NYHA FC III/IV < 50%	4	1.08 [1.02–1.15]	0.01*	86.21
WHO FC III/IV ≥ 50%	2	1.07 [0.95–1.22]	0.28	92.21
WHO FC III/IV < 50%	2	1.10 [1.06–1.14]	< 0.001*	0.04
Group 1 PH only	1	1.15 [1.07–1.23]	< 0.001*	–
Group 3 and 4 PH	3	1.06 [1.00–1.13]	0.05	86.59
mPAP ≥ 50 mmHg	1	1.01 [0.99–1.03]	0.19	–
mPAP < 50 mmHg	3	1.11 [1.07–1.14]	< 0.001*	2.34
**RIMP**	**6**	**1.51 [1.23**–**1.86]**	< 0.001*	**28.02**
NYHA FC III/IV ≥ 50%	1	2.06 [1.16–3.67]	0.01*	–
NYHA FC III/IV < 50%	5	1.46 [1.16–1.83]	< 0.001*	30.94
WHO FC III/IV ≥ 50%	3	1.47 [1.00–2.16]	0.05	54.51
WHO FC III/IV < 50%	3	1.58 [1.36–1.84]	< 0.001*	0
Group 1 PH only	3	1.56 [0.91–2.67]	0.11	57.54
Group 3 and 4 PH	3	1.58 [1.36–1.82]	< 0.001*	0
mPAP ≥ 50 mmHg	4	1.54 [1.07–2.22]	0.02*	46.57
mPAP < 50 mmHg	2	1.57 [1.35–1.83]	< 0.001*	0

Bold shows the RV fuctional parameters assessed, and the below each parameters are the subgroups of the parameters

HR, Hazard ratio; TAPSE, Tricuspid Annular Plane Systolic Excursion; RVSP, Right Ventricular Systolic Pressure; RVLS, Right Ventricular Longitudinal Strain; RVFAC, Right Ventricular Fractional Area Change; RVEF, Right Ventricular Ejection Fraction; RIMP, Right Ventricular Index of Myocardial Performance; NYHA, New York Heart Association; WHO, World Health Organization; FC, functional class; PH, pulmonary hypertension; mPAP, mean pulmonary artery pressure

*Shows significance of < 0.05

### Summary receiver operating characteristics curves of TAPSE and RVLS

**Fig. 3:**
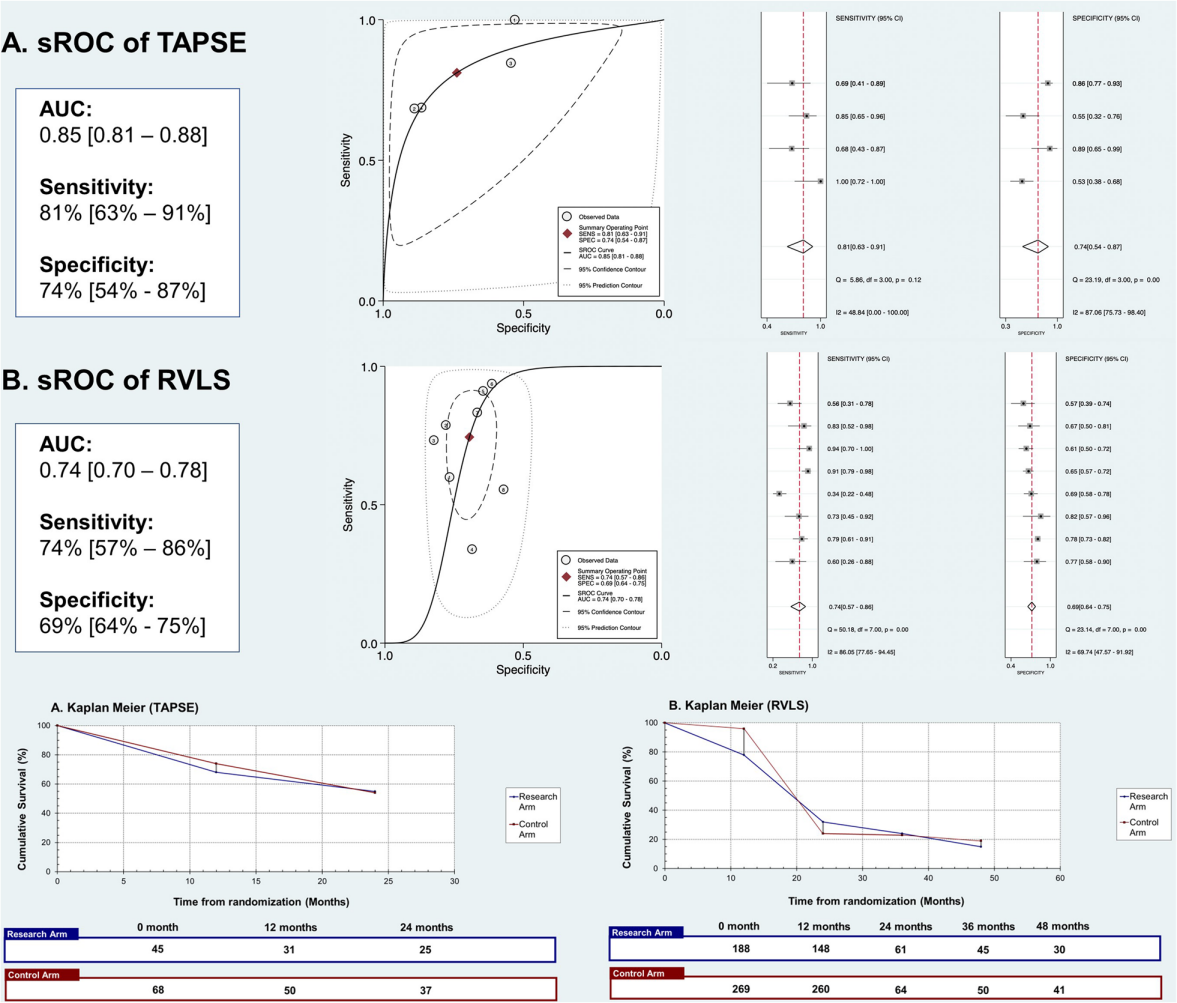
Summary receiver operating characteristic curves, pooled sensitivity, specificity, and Kaplan–Meier survival analysis of **A** TAPSE and **B** RVLS. Abbreviations: 95% CI, 95% confidence interval; AUC, area under the curve; RVLS, Right Ventricular Longitudinal Strain; SENS, Sensitivity; SPEC, Specificity; sROC, Summary Receiver Operating Characteristic; TAPSE, Tricuspid Annular Plane Systolic Excursion

In pursuit of evaluating the predictive capacity of RV functional parameters concerning mortality in precapillary PH, the utilization of sROC curves was employed, as depicted in Fig. 3. The summarized ROC curve provided an overarching perspective of predictive accuracy, effectively encapsulating the delicate balance between sensitivity and specificity. In this regard, the calculated AUC for TAPSE was found to be 0.85 [0.81–0.88], signifying a commendable level of discriminatory power. TAPSE exhibited a sensitivity of 0.81 [0.63–0.91] coupled with a specificity of 0.74 [0.54–0.87], reinforcing its notable prognostic value. Conversely, for RVLS, the derived AUC amounted to 0.74 [0.70–0.78], implying a moderately robust discriminatory capability. RVLS was associated with a sensitivity of 0.74 [0.57–0.86] and a specificity of 0.69 [0.64–0.75].

### Kaplan–Meier survival analysis of TAPSE and RVLS

The Kaplan–Meier curves illustrating the estimated composite mortality outcomes are thoughtfully depicted in Fig. 3, stratified by TAPSE and RVLS. The duration of survival analysis for TAPSE was restricted to a maximum of 24 months, whereas studies assessing RVLS extended their data coverage to 48 months. Remarkably, the curves unveiled a compelling trend: Lower TAPSE values and higher RVLS values were conspicuously associated with diminished cumulative survival rates in contrast to their respective counterparts. This underscores the potential prognostic significance of these parameters in gauging the mortality risk for individuals grappling with precapillary PH.

## Discussion

To the extent of our knowledge, this is the first systematic review and meta-analysis that reports the significance of various right ventricular echocardiographic parameters as prognostic tools among a relatively large cohort of precapillary PH patients. It is also evident that TAPSE, RVLS, RVFAC, RVEF, and RIMP all emerge as reliable predictors of mortality based on our analysis of pooled HR from the studies. In precapillary PH, survival is contingent on right ventricular dysfunction, a consequence of increased pulmonary vascular resistance. The compensatory mechanisms of the heart, such as hypertrophy or increased contractility, become maladaptive, ultimately failing to counteract the elevated pressure load [32].

### TAPSE

TAPSE involves the use of an M-mode ultrasound technique within an apical four-chamber view to assess the longitudinal displacement of the tricuspid ring in the right ventricle. Our analysis has shown that TAPSE is markedly reduced in patients with precapillary PH. This observation aligns with the research conducted by Forfia et al., and a TAPSE lower than 1.8 cm is associated with a lower right ventricular function [15]. This result may be attributed to the increased right ventricular pressure load, which leads to a reduced level of systolic excursion and, consequently, a decrease in ejection fraction [33]. Our analysis demonstrated a robust link between decreased TAPSE and mortality in precapillary PH patients, indicating a 38% higher hazard of mortality in individuals with PH. This includes a comprehensive sROC analysis of TAPSE, emphasizing its prognostic value with 81% sensitivity and 74% specificity. Kaplan–Meier survival analysis over twelve months highlighted TAPSE as the most significant prognostic factor. This finding is consistent with the results of a meta-analysis conducted by Meijerink et al., which similarly demonstrated a correlation between reduced TAPSE and increased mortality [34]. In addition, a recent meta-analysis by Baggen et al. also established decreased TAPSE as a reliable prognostic tool in patients with PH [35]. Interestingly, a study identified a nonsignificant association between TAPSE and all-cause mortality [9]. Despite unclear contributors to this disparity in consolidated findings, subgroup analysis consistently identifies TAPSE as a reliable prognostic tool across subgroups. Emphasizing TAPSE's clinical significance, this echoes its crucial role in predicting adverse outcomes, particularly mortality, in precapillary pulmonary hypertension, while evaluating right ventricular function.

### RVSP

RVSP, a crucial echocardiographic parameter for PH screening, estimates PASP by measuring tricuspid regurgitant jet velocity. Our analysis showed a substantial RVSP increase in PH patients (mean value: 77.5 mmHg). Bossone et al. established a key PASP cutoff (36 mmHg), highlighting a significant RVSP disparity between normal individuals and those with PH [36]. Furthermore, a cohort study conducted by Strange et al. found that a RVSP over 30 mmHg was significantly associated with increased 1-year and 5-year mortality [36]. RVSP's potential as a diagnostic tool for pulmonary hypertension is noted. Despite reported high sensitivity and specificity, its inconsistencies imply unreliability as a diagnostic parameter. Surprisingly, our analysis found no statistically significant association between RVSP and mortality, contrary to Corciova et al.'s study, which reported a significant correlation between PASP and mortality [37]. In a recent meta-analysis conducted by Prosperi-Porta et al., it was observed that a 10 mmHg increment in PASP is associated with a 1.5-fold increase in the likelihood of adverse events [38]. Conversely, multiple studies have indicated that the ratio of TAPSE, RVEF, or RVFAC to PASP represents a more dependable prognostic tool for predicting mortality in patients with PH. This is attributed to the capacity of right ventricular–arterial coupling to effectively reflect right ventricular afterload and contractility [6, 39]. These findings suggest that the use of multiple echocardiographic parameters may improve the predictive value for adverse outcomes and mortality in patients with PH. Notably, within our subgroup analysis, we found a strong correlation between RVSP and patients with PH categorized as the WHO FC III/IV. In the advanced stages of PH, patients face an elevated risk of experiencing adverse outcomes as a consequence of their hemodynamic instability [40, 41]. This instability, driven by vascular changes and impaired pulmonary compliance, leads to elevated resistance, straining the right ventricle, causing systemic congestion and inflammation, contributing to higher mortality in advanced PH.

### RVLS

The well-established link between reduced RVLS and higher mortality in PH patients establishes RVLS as a valuable mortality prognostic tool. Calculated by averaging strain in six right ventricular segments, RVLS reflects both global and regional systolic function in the context of PH [42]. Through our analysis, it was evident that patients with PH have a higher or less negative RVLS value compared to normal patients with a mean value of − 16.78%. This is attributed to the increased right ventricular mechanical stress and increased contractile required for effective ejection in PH patients [32]. Our analysis has revealed that an elevated RVLS is linked to a 74% increased risk of mortality, with corresponding sensitivity and specificity values of 74% and 69%. These findings suggest that when compared to TAPSE, RVLS may have a comparatively lower significance as a prognostic factor in patients with precapillary PH. Similar to TAPSE, the disparity in survival between patients with higher RVLS and the control group is most pronounced at the twelve-month mark. A meta-analysis by Shukla et al. reported a significantly higher HR of 3.78 in patients with an RVLS of > − 20% [43]. The differences in findings might be attributed to the use of a specific cutoff value, a criterion that we did not utilize in our analysis. In addition, our finding is consistent with a study conducted by Focardi et al., which highlighted that RVLS can predict right ventricular performance, thus playing a crucial role in determining the mortality risk among individuals with PH [44]. Other studies have also reported positive correlation between RVLS and all-cause mortality [45, 46]. Ultimately, this meta-analysis further strengthens the existing body of literature surrounding the utilization of RVLS as a prognostic parameter in individuals with PH.

### RVFAC

RVFAC is an index of global systolic performance and contractility, as it measures the percentage of change in the area of right ventricle during systole and diastole [42]. We found that RVFAC is markedly diminished in PH patients with a mean value of 29.81%. Nonetheless, as indicated by a study by Slegg et al., it is important to note that the normal reference values for RVFAC differ by gender, with a threshold of < 30% for males and < 35% for females. Moreover, RVFAC interestingly exhibits a statistically significant reduction in patients diagnosed with precapillary PH [47]. In PH, elevated pulmonary vascular resistance increases the right ventricular afterload, demanding a higher level of contractility to maintain cardiac output. However, this adaptation may become maladaptive over time, leading to a decrease in RVFAC. A reduction in RVFAC indicates the right ventricle's inability to effectively contract, contributing to a poor prognosis. The results of our analysis revealed a strong association between reduced RVFAC and mortality in precapillary PH patients with an increased risk of mortality by 40%. Teramoto et al. conducted a study, reporting a noteworthy correlation between an RVFAC value < 25.5% and all-cause mortality in patients with PH [48]. Nevertheless, further investigations are warranted to establish the precise cutoff value that consistently holds prognostic significance. In patients with WHO FC III/IV < 50%, Group 1 PH, and mPAP ≥ 50 mmHg, RVFAC exhibited heightened prognostic significance.

### RVEF

The assessment of RVEF involves calculating the volume of blood expelled from the right ventricle during systole compared to the total end-diastolic volume [42]. In our analysis, we observed the prognostic significance of RVEF in a specific subgroup, specifically those in which less than 50% of the patients were classified under WHO FC III/IV. This result may be due to the advanced disease state in this subgroup, where right ventricular dysfunction plays a more critical role in prognosis. A very recent meta-analysis by Kitano et al. has reported that a pooled HR of 2.54 for RVEF in patients with PH [49]. This result contradicts our analysis, which did not show significant prognostic value for RVEF in the overall cohort. The disparity in findings underscores the complexity of RVEF's role in predicting mortality in PH and may be attributed to variations in patient characteristics, PH etiologies, and methods of RVEF measurement. Therefore, comprehensive research which includes diverse patient populations is necessary to establish a clearer understanding of its prognostic utility in the context of PH.

### RIMP

Another significant echocardiographic parameter is the RIMP, also known as the Tei Index or Myocardial Performance Index. These terms are frequently used interchangeably to refer to the same parameter. Through our analysis, it was demonstrated that RIMP holds prognostic significance, particularly among patients with advanced PH in WHO FC III/IV. Interestingly, RIMP also showed superior predictive capacities of mortality within Group 3 and 4 PH, underlining its significance as a prognostic parameter. Similar with previous parameters discussed, this result may also be attributed to the increased hemodynamic instability often experienced by patients in later stages of PH. Studies surrounding the use of RIMP as a standalone parameter in predicting mortality is very limited. Intriguingly, research conducted by Grapsa et al. found that RIMP serve as a valuable prognostic factor in individuals with PH [50].

### Study limitations and recommendations

The limitations of our meta-analysis should be considered. Due to the restricted availability of complete data from the included studies, our investigation used a subset of data for sROC and Kaplan–Meier analyses. The data pertaining to PVR values are absent in most studies. Although PVR is crucial in the context of precapillary PH, the lack of data precludes analysis. PVR serves as a critical measurement in differentiating between precapillary, postcapillary, and combined capillary PH. Additionally, the majority of the studies in our analysis were single-center observational retrospective in nature, which can introduce potential biases related to data collection and patient selection. Future research involving randomized controlled trials would provide a more reliable foundation for analysis. Furthermore, the lack of detailed patient-level data regarding NYHA and WHO functional class led us to determine 50% as an approximation for the majority, which might not precisely represent the entire spectrum of functional class distribution. Hence, conducting further research to address these limitations would not only enhance the precision of the results but also increase their clinical relevance and applicability in patients with PH.

## Conclusions

In conclusion, right ventricular echocardiographic functional parameters, such as TAPSE, RVLS, RVFAC, RVEF, and RIMP, are substantial prognostic markers for precapillary PH mortality. The predictive value is influenced by disease severity, etiology, and mPAP. While these findings offer valuable insights, further research, including RCTs and patient-level data, is needed to enhance accuracy and clinical applicability in PH patients.

### Supplementary Information

Additional file 1.

## Data Availability

Data available within the article. The authors confirm that the data supporting the findings of this study are available within the article.

## Abbreviations

6MWD: 6-Minute walking distance
CTEPH: Chronic thromboembolic pulmonary hypertension
FC: Functional class
mPAP: Mean pulmonary arterial pressure
NYHA: New York Heart Association
PH: Pulmonary hypertension
PWP: Pulmonary wedge pressure
RHC: Right heart catheterization
RIMP: Right Ventricular Index of Myocardial Performance
RVFAC: Right Ventricular Fractional Area Change
RVEF: Right Ventricular Ejection Fraction
RVLS: Right Ventricular Longitudinal Strain
RVSP: Right Ventricular Systolic Pressure
TAPSE: Tricuspid Annular Plane Systolic Excursion
