# Supplementary material for: Prognostic values of right ventricular echocardiography functional parameters for mortality prediction in precapillary pulmonary hypertension: a systematic review and meta-analysis
Source: Egypt Heart J. 2024 Aug 14;76:105. doi: 10.1186/s43044-024-00539-6 (PMC11324623; doi:10.1186/s43044-024-00539-6)
Supplement: Supplementary file 1 — Additional file 1. [file 43044_2024_539_MOESM1_ESM.docx]

**
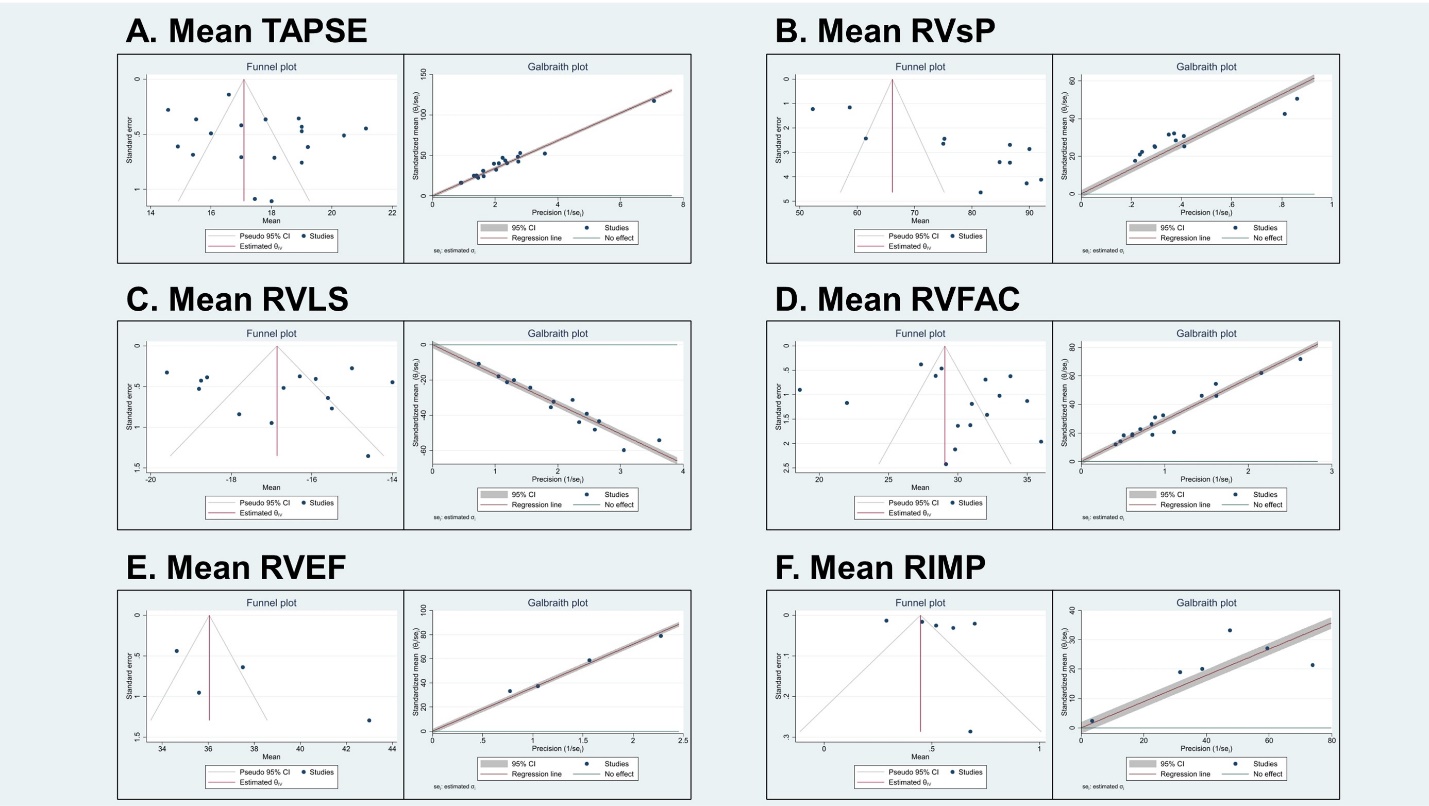

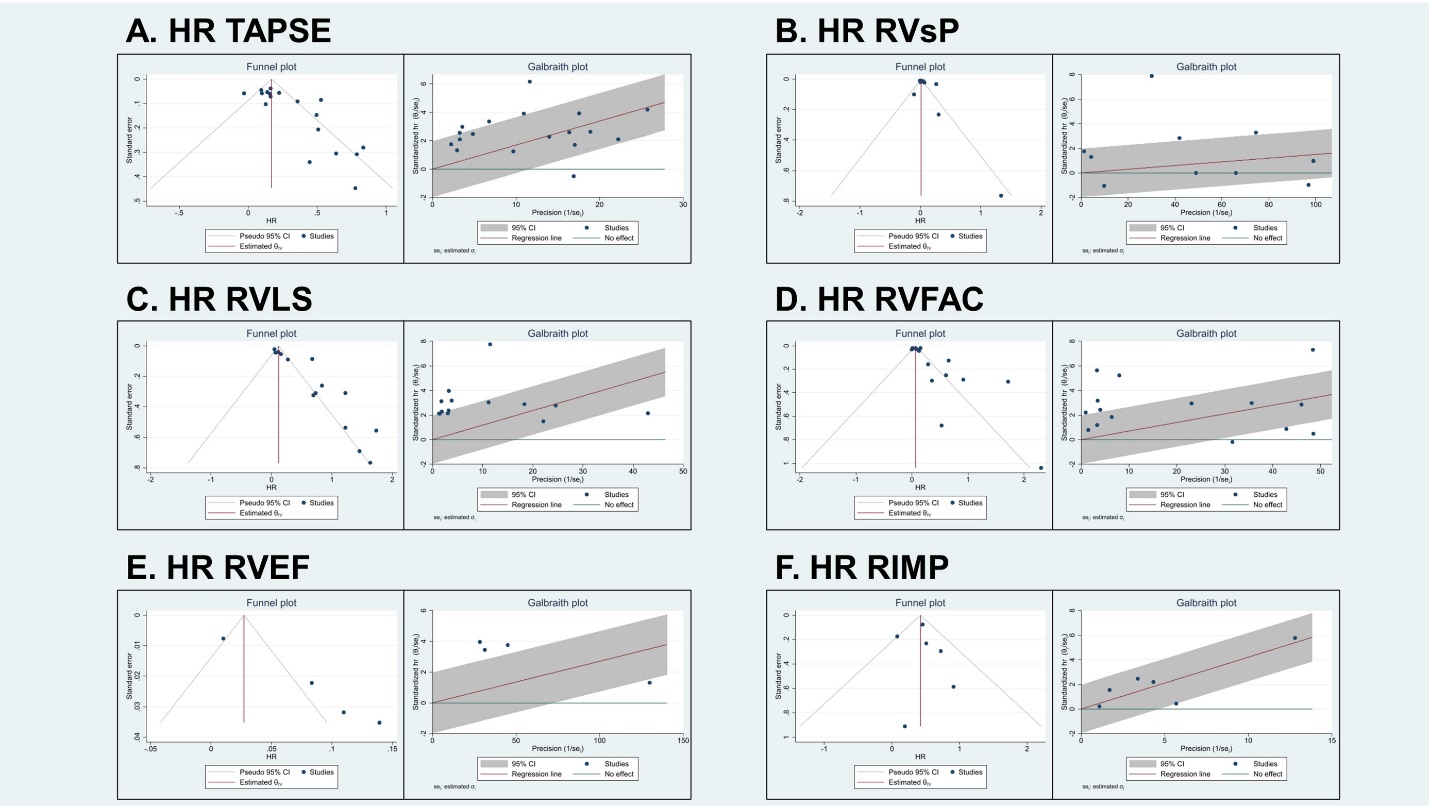
Supplementary File 1**

Supp Figure 1. Funnel and Galbraith plots of mean right ventricular functional parameters

Supp Figure 2. Funnel and Galbraith plots of hazard ratios right ventricular functional parameter

Supp Table 1. Quality assessment of included studies

| **Author, year** | **Selection** | | | | **Comparability** | **Outcome** | |  | **Total Score** |
| --- | --- | --- | --- | --- | --- | --- | --- | --- | --- |
|  | **Representativeness of exposed cohort** | **Selection of unexposed cohort** | **Ascertainment of exposure** | **Outcome was not present at start of study** | **Comparability of cohorts on the basis of the design or analysis** | **Assessment of outcome** | **Follow-up length** | **Adequacy of follow-up** |  |
| Badagliacca et al, 2016 | * | * | * | * | * | * | * |  | 7 |
| Butcher et al. 2022 | * | * | * | * | * | * | * |  | 7 |
| Chen et al, 2017 | * | * | * | * | * | * | * |  | 7 |
| Ciarka et al, 2010 | * | * | * | * | * | * | * | * | 8 |
| da Costa et al, 2017 | * | * | * | * | * | * | * | * | 8 |
| Dandel et al, 2014 | * | * | * | * | * | * | * |  | 7 |
| Fine et al, 2013 | * | * | * | * | * | * | * | * | 8 |
| Forfia et al, 2006 | * | * | * | * | * | * | * |  | 7 |
| Ghio et al, 2010 | * | * | * | * | * | * | * |  | 7 |
| Giusca et al, 2013 | * | * | * | * | * | * | * |  | 7 |
| Greiner et al, 2018 | * | * | * | * | * | * | * | * | 8 |
| Haddad et al, 2015 | * | * | * | * | * | * | * | * | 8 |
| Haeck et al, 2012 | * | * | * | * | * | * | * | * | 8 |
| Hulshof et al, 2021 | * | * | * | * | * | * | * | * | 8 |
| Ishii et al, 2023 | * | * | * | * | * | * | * |  | 7 |
| Li et al, 2021 | * | * | * | * | * | * | * | * | 8 |
| Mahapatra et al, 2006 | * | * | * | * | * | * | * |  | 7 |
| Mathai et al, 2011 | * | * | * | * | * | * | * |  | 7 |
| Moceri et al, 2017 | * | * | * | * | * | * | * | * | 8 |
| Murata et al, 2016 | * | * | * | * | * | * | * |  | 7 |
| Park et al, 2015 | * | * | * | * | * | * | * |  | 7 |
| Raymond et al, 2002 | * | * | * | * | * | * | * | * | 8 |
| Sachdev et al, 2011 | * | * | * | * | * | * | * |  | 7 |
| van Kessel et al, 2016 | * | * | * | * | * | * | * |  | 7 |

Abbreviations: ASPECTS, Alberta Stroke Program Early Computed Tomography Score; NIHSS, National Institutes of Health Stroke Scale
